# Non-cytotoxic functions of CD8 T cells: “repentance of a serial killer”

**DOI:** 10.3389/fimmu.2022.1001129

**Published:** 2022-09-12

**Authors:** Mouhamad Al Moussawy, Hossam A. Abdelsamed

**Affiliations:** ^1^ Department of Surgery, School of Medicine, University of Pittsburgh, Pittsburgh, PA, United States; ^2^ Starzl Transplantation Institute, School of Medicine, University of Pittsburgh, Pittsburgh, PA, United States; ^3^ Pittsburgh Liver Research Center, School of Medicine, Pittsburgh, PA, United States

**Keywords:** non-cytotoxic, cross-talk, direct-, indirect-, CD8 T cells (CTLs)

## Abstract

Cytotoxic CD8 T cells (CTLs) are classically described as the “serial killers” of the immune system, where they play a pivotal role in protective immunity against a wide spectrum of pathogens and tumors. Ironically, they are critical drivers of transplant rejection and autoimmune diseases, a scenario very similar to the famous novel “*The strange case of Dr. Jekyll and Mr. Hyde”.* Until recently, it has not been well-appreciated whether CTLs can also acquire non-cytotoxic functions in health and disease. Several investigations into this question revealed their non-cytotoxic functions through interactions with various immune and non-immune cells. In this review, we will establish a new classification for CD8 T cell functions including cytotoxic and non-cytotoxic. Further, we will discuss this novel concept and speculate on how these functions could contribute to homeostasis of the immune system as well as immunological responses in transplantation, cancer, and autoimmune diseases.

## 1 A brief history of CD8 T cell cytotoxicity

The history of science is full of discoveries which usually begins with the intention to understand a specific phenomenon in a cohort of patients or preclinical disease models- so called “phenomenology”; however, most of the time, it ends with a completely different story. For instance, much of what we know about cell-mediated cytotoxicity was borne out from pioneering *in vitro* studies started in the early 1960s, which investigated graft rejection using animal models. Indeed, the first report demonstrated that cellular antibodies i.e., lymphocytes from canines transplanted with homograft kidney destroy allogeneic targets *in vitro* as observed microscopically (1). Along the same lines, lymphocytes from Balb/c mice allosensitized with C3H cells were shown to target and induce C3H cytolysis (2). Similarly, thoracic duct or lymph node cells from allosensitized rodents were able to target and kill donor kidney cells *in vitro* (3).

The above-mentioned observations further served as an impetus to define the nature of these cells exerting cytotoxic killing. By the end of 1960s and early 1970s, a series of elegant studies demonstrated that treatment of such populations with Thy1 (CD90), Ly-2 (CD8a), and Ly-3 (CD8b) anti-sera abolished the anti-allogeneic cytotoxicity of mouse cells, suggesting the cytotoxic effect of T cells (4–6). However, the mechanisms of cytotoxicity and T cell specificity were not clear at that time. It was not until D. B. Amos hypothesized that cytotoxicity was a result of a two-step process: (1) specific recognition followed by (2) non-specific cytotoxicity, which implied that there should be a specific T cell receptor for antigen recognition (7). Later, several lines of evidence supported this notion showing that sensitized lymphocytes isolated from allo-immunized mice showed both specificity and cytotoxicity against their targets. In these studies, upon culturing these cells with macrophage monolayers expressing the allo-H2 MHC antigen, the non-adherent cells did not possess a cytotoxic activity while the adsorbed cells showed cytotoxicity when eluted from the monolayer macrophage cells. These data suggested that the cytotoxic cells were adsorbed on the monolayers because they express receptors that could recognize the H2 alloantigen (8–10). In two seminal papers, Zinkernagel and Doherty further refined the specificity of the lymphocyte receptor binding to their target cells showing MHC restriction using LCMV-infected mouse model. They proposed an “altered-self or the one receptor model” where MHC recognition occurs *via* T cell receptor rather than the “two-receptor or intimacy model” in which MHC recognition is a separate event from viral antigen recognition by the T cell receptor (11, 12).

During this period, huge strides had been achieved in understanding CD8 T cell (CTL) biology including the nature of the cytotoxic cells and antigen recognition by receptor; albeit the mechanism(s) involved in cell-mediated killing post-antigen recognition were still enigmatic. It all began with C. Sanderson’s observation where the dying target cell showed morphological changes that was distinct from complement-mediated lysis but similar to recently described apoptotic cell death (13). At that time, it has become appreciated that CTLs are able to lyse several targets sequentially- so called “serial killing” (14–16).

**Figure 1:**
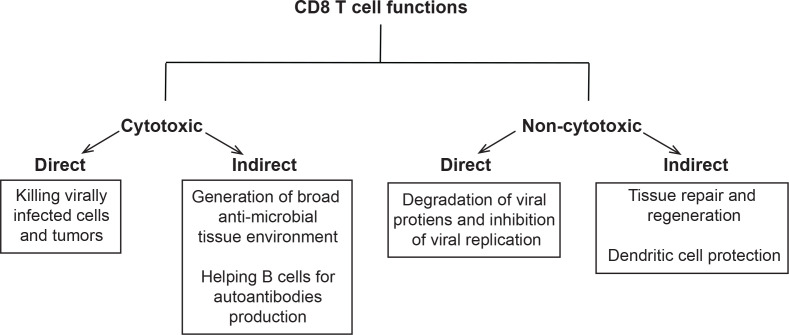
Classification of CD8 T cells functions: Schema showing classification of these functions into cytotoxic and non-cytotoxic. The cytotoxic functions were further partitioned into direct (killing tumors or virally infected cells) and indirect (recruitment of immune cells to the site of infection, tumor vaccines, and B cell help in cancer, pathogen clearance and autoimmunity). The non-cytotoxic functions were classified into direct (degradation of viral proteins and inhibition of viral replication) and indirect (tissue repair and regeneration, protection of DCs, and homeostasis).

Despite their well-documented cytotoxic capabilities, several elegant studies emerged in the past 30 years showing that CTLs are equipped with non-cytotoxic functions as well. These cytotoxic and non-cytotoxic functions can be exerted directly through the killing machinery or indirectly *via* cross-talk with other immune cells and possibly non-immune cells. Hence, we thought to classify CTLs functions into four types: (1) Direct cytotoxicity, (2) Indirect cytotoxicity, (3) Direct non-cytotoxicity, and (4) Indirect non-cytotoxicity **(** **Figure 1** **)**.

## 2 Cytotoxic and non-cytotoxic functions of CD8 T cells

### 2.1 Direct cytotoxicity: A serial killer with many weapons

**Figure 2:**
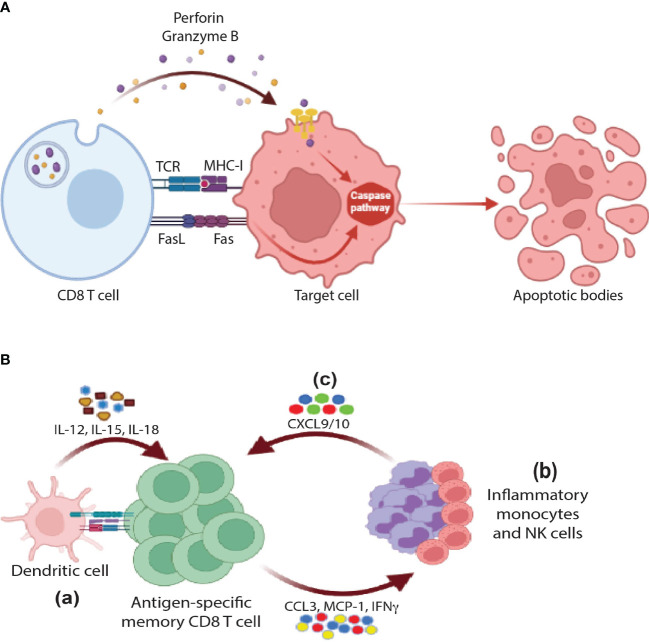
Cytotoxic functions of CD8 T cells: **(A)** Direct cytotoxicity: CD8 T cells release perforin and granzyme B through MHC-I/TCR axis in order to activate the apoptotic pathway in the target cells. Further, the interaction between FasR and FasL results in activation of caspases and eventually apoptosis **(B)** Indirect cytotoxicity: (a) In response to activation by antigen re-exposure and cytokine release, antigen-specific memory CD8 T cells release a wide range of cytokines and chemokines such as IFNγ, CCL3, and MCP1 that would help (b) recruit innate cells such monocytes and NK cells that in turn secrete CXCL9/10 in order to further (c) amplify the recruitment of memory CD8 T cells, activate of B cells, and DCs (not shown in the figure).

One of the cardinal features of CTLs is their potent killing capacity against target cells including virally infected cells as well as tumor cells. They perform these functions directly using a whole arsenal of effector molecules including granzymes, perforin, and FAS/FASL pathway **(** **Figure 2A** **).**

The cytotoxic effect of these molecules was first hinted by the observation of microscopical tubular lesions in target cell membranes (perforated cell membrane) following incubation with cytotoxic T cells or granules isolated from them (17–21). Consequently, a protein homologous to C9 of the complement system was discovered and isolated from these granules known as perforin. Both proteins can polymerize and form the membrane attacking complex resulting in membrane perforation (22–26). Along the same lines, granule exocytosis model was proposed around the same time hypothesizing that granule contents including perforin were released by exocytosis at the synaptic space between CTL and target cell. To draw a cause-and-effect relationship, several studies showed marked decrease in the cytotoxicity of CTLs isolated from perforin knockout mice or transfected with perforin siRNA, implying the importance of such molecule in cytotoxicity (27–29).

However, researchers began to realize that other effector molecules beside perforin could also induce cytotoxicity since CTLs can activate apoptosis as observed microscopically while purified perforin induced necrosis (15, 30). Hence these observations hinted that other effector molecules could work hand-in-hand with perforin resulting in cell-mediated cytotoxicity through granule exocytosis mechanism. One of the strong candidates were serine esterases since cell-mediated cytotoxicity was blocked in the presence of their inhibitors (31, 32). Later on, they were named as granzymes since they could be isolated from granules (33–35). Consequently, perforin-granzyme pathway was considered as one of the major mechanisms of cell-mediated cytotoxicity where perforin opened pores in the target cell membrane facilitating the entry of granzymes into the cytosol including granzyme B (GzmB), which in turn directly initiated apoptosis through activation of Caspase 3 or indirectly through interaction with BH3-interacting domain death agonist (BID) (30, 36, 37).

Although the perforin-granzyme pathway is considered as one of the major pathways that CTLs use in their killing process, it still does not fully account for the CTL killing capacity. For instance, lymphocytes that lack perforin were still cytotoxic. Furthermore, although granule exocytosis requires calcium signaling, CTLs are still capable of killing their targets in a calcium-independent manner (38–40). These observations along with other studies suggested the existence of an alternative cytotoxic killing machinery, leaving the field in a big debate (41). The discovery of the second cytotoxicity pathway started with the generation of T cell hybridoma PC-60-d10S showing calcium independent as well as non-MHC restricted killing capacity specially against thymocytes (42, 43). Around the same period, Nagata’s lab reported that thymocytes isolated from wild-type mice expressed CD95 (APO-1/Fas), a known cell death containing domain receptor, while *lpr* mice did not (44) (mice with CD95 mutation leading lymphoproliferation phenotype). Later on, the same lab was successful to clone the ligand using Fas-Fc construct to select and isolate PC60-d10S clones expressing the Fas ligand (FasL/CD95L) using FACS (45). In the FAS-mediated mechanism, the binding of FASL to FAS expressed by the target cell result in activation of Caspase 8 through FAS-associated death domain protein (FADD), which ultimately results in activation of Caspase 3 and induction of apoptosis (46, 47) **(** **Figure 2A** **)**. Thus far, the above-mentioned studies demonstrated that CTLs kill their target cell through two main pathways: (1) perforin-granzyme granule exocytosis mechanism and (2) FAS-dependent pathway. However, several studies showed that CTLs can also contribute to the process of cytotoxicity indirectly through cross-talk with other immune cell types. This type of cytotoxicity will be discussed in the following section.

### 2.2 Indirect cytotoxicity: Calling for help

#### 2.2.1 Tissue-resident broad anti-microbial state

Since the immune system is constituted of multiple cell types, it is expected that different cells cross-talk to each other to perform specific functions. As discussed in the previous section, CTLs can execute their killing functions locally through direct contact with target cells in an MHC-I dependent manner using a wide-spectrum of effector molecules **(** **Figure 2A** **)**. However, they should be present in sufficient numbers at peripheral tissues to control the pathogen, which is not the case prior to infections. To circumvent such dilemma, following infection, T cells migrate to non-lymphoid tissues and differentiate into tissue-resident non-circulating memory T cells (T_RMs_). Both antigen presentation and cytokines are required for the differentiation of T_RMs_. For instance, in mice, naïve cells require cross-talking to DNGR-1^+^ dendritic cells (cDC1, CD103^+^ CD8a^+^ DCs) for the generation of T_RMs_ in response to Flu and Vaccinia viruses (48, 49). This type of communication also involves IL-12, IL-15, and CD24 co-stimulation signals as well (50–54). In humans, the cross-talk of CD1c^+^ DCs with naïve CD8 T cells plays a pivotal role in generation of T_RMs_ in a TGF-β-dependent manner (55). Further, both effector T cells (T_EFF_) and central memory T cells (T_CM_) have the capacity to differentiate into T_RMs_ (56). However, the question remains: “how such small numbers of T_RMs_ still can control pathogen dissemination”.

One way to overcome this challenge is to start communicating with other immune cell types. As shown in **Figure 2B**, upon antigen and cytokine stimulation, memory T cells rapidly express IFNγ and chemokines promoting recruitment and activation of innate myeloid and lymphoid cells including monocytes and NK cells. These cells further amplify the recruitment of memory cells *via* expression of CXCL9/10 chemokines resulting in the generation of systemic and/or tissue broad anti-microbial state **(** **Figure 2B** **)**. The Masopust lab and others spearheaded elegant studies to examine this model (57–61). For example, Schenkel et al. showed that T_RMs_ were able to recruit bystander circulating memory CD8 T cells to peripheral tissues through VCAM-1/IFNγ axis by using an OT-I or P14 chimeric mouse model. In this model, naïve OT-I or P14 CD8 T cells were adoptively transferred to B6 mice followed by VV-OVA or LCMV infection respectively. To activate T_RMs_ at the female reproductive tract (FRT), OVA or LCMV-specific peptides were injected transcervically (t.c). Concurrently, there was an upregulation of VCAM-1 (a4β1-CD49d) on vascular endothelium and IFNγ by reactivated T_RMs_. These events were associated with recruitment of OT-I specific CD8 T cells (bystander) in response to LCMV infection, which was blocked by neutralization of VCAM-1 or IFNγ. Further, the recruitment of additional immune cell types, including B cells, to FRT as well as the activation of innate cells such as DCs and NK cells were observed (57, 58). Along the same lines, it has been shown by Soudja et al. that memory CD4 and CD8 T cells plays an essential role in orchestrating activation of splenic innate immune cells following secondary infections in an IFNγ dependent manner (60). In conclusion, T_RMs_ can deploy their cytotoxicity indirectly through recruitment of a wide-range of immune cell types acting as guardians of peripheral tissues in case of reinfection.

#### 2.2.2 Tumor vaccines

Indirect cytotoxicity has been further supported by other studies using tumor vaccine mouse models. For example, Kalinski’s lab demonstrated that CD8 T cells can act as *de fecto* helper cells supporting an effective anti-tumoral DC immunological response (62). In these studies, they showed that the adoptive transfer of autologous DCs loaded with poorly immunogenic MC38 tumor lysate to the animals bearing the same tumor was only marginally effective. However, the inclusion of OVA257–264 epitope into the vaccine supported the generation of MC38-specific CTL responses in wild-type B6 animals and tumor clearance. These data suggest that non-specific CD8 T cells could play an indirect cytotoxic role through harnessing the killing capacity of antigen-specific T cells *via* unknown mechanism(s). Similar data were also obtained in a model of wild-type mice harboring memory responses against LCMVgp33–41, a dominant epitope of a natural mouse pathogen, where the inclusion of LCMVgp33–41 peptide strongly enhanced the induction of CTLs against MC38 tumors. These vaccines not only elevated CTLs’ function against the poorly immunogenic MC38 adenocarcinoma but also against the highly immunogenic OVA-expressing EG7 lymphoma (62). These studies provide a mechanistic insight into the role of memory CD8 T cells in enhancing the anti-tumoral effect, which suggest indirect cytotoxic function. However, additional studies are required to determine which T cells are responsible for the killing of the tumor, is it the tumor-specific, non-specific (bystander), or both?

#### 2.2.3 Helper function towards B cells

The helper function assumed by T cells has been extended to encompass the role of CD8 T cells in inducing antibody production by B cells and their involvement in killing tumors, pathogen clearance and autoimmunity. More than 30 years ago, the Le Gros lab showed that polyclonal stimulation (PMA/Ionomycin + IL-2 + IL-4) of total CD8 T cells isolated from murine lymph nodes (LNs) resulted in (1) down regulation of CD8α, (2) decrease in cytotoxicity, (3) downregulation of IFNγ and perforin, (4) upregulation of T_H_2 cytokines IL-4, IL-5, and IL-10, and (5) help for B cells to produce IgG antibodies (63). The authors took their analyses one step further and examined whether this phenomenon was MHC-I restricted. Indeed, stimulating CD8 T cells with MHC allo-antigens in the presence of IL-4 resulted in non-cytolytic phenotype (63). Co-culturing these activated cells with autologous B cells resulted in the secretion of IgG antibodies in the culture supernatant. This early study put CD8 T cells at a crossroad with antibody producing B cells, which underlined a possible indirect cytotoxic role of CD8 cells in the pathogenesis of autoimmunity.

Later on, extensive body of literature discussed the existence of T follicular helper CD4 cells (CD4 Tfh cells) and their role in providing help to B cells for antibody production (64–70). Similar to CXCR5^+^ PD1^+^ CD4 Tfh cells, CXCR5^+^ CD8 T cells exhibit a B cell helper function, where they support antibody production either (1) through a direct interaction with B cells (71–74) or (2) *via* enhancement of CD4 T cell-B cell interaction (75). Indeed, upon TCR stimulation, these cells upregulate CD70, OX40 and ICOS molecules, which are required for T cell dependent humoral responses.

The indirect cytotoxic function of CXCR5^+^ CD8 T cells in antibody production and B cell support had been demonstrated in various disease states. For example, in gastric cancer, the accumulation of CXCR5^+^ CD8 T cells in the tumor is associated with better patient overall survival (OS) (76). Similarly, IL-21 producing CXCR5^+^ CD8 T cells accumulate in the hepatocellular carcinoma tumor tissues in close proximity to CD19^+^ B cells, which predicts better disease prognosis (71). These studies raise the question: what type of cross-talk is taking place within the tumor microenvironment. One can predict interaction between B cells and CXCR5^+^ CD8 T cells. Indeed, co-culturing these cells with B cells resulted in enhanced *in vitro* differentiation of B cells as well as an increase in IgG and reduction in IgM production. Hence, the indirect cytotoxic role of CD8 T cells against tumors could be explained by helping B cells to produce antibodies, which in turn bind tumor cells and recruit NK cells to initiate antibody-dependent cell cytotoxicity (ADCC). In another study, CXCR5^+^ ICOS^+^ CD8 T cells had been shown to infiltrate tumoral lymph nodes (LNs) in Hodgkin lymphoma (HL) (77). This subpopulation was shown to upregulate the expression of IL-2, IL-4 and IL-21, key cytokines for antibody production and B cell support. However, they showed weak expression of effector molecules including GzmB, perforin, and IFNγ. Similarly, CXCR5^+^ PD-1^+^ ICOS^+^ CD8 T cells isolated from nasal polyp tissue promote antibody production when co-cultured with B cells (78). Along the same lines, the Youngblood lab showed elegantly that HIV-specific CD8 T cells isolated from Elite controllers (ECs) expressed high levels of CXCR5 transcript compared to ART-suppressed non-controllers (79), which suggested the protective role of CXCR5^+^ CD8 T cell in EC patients. Further, the *in vitro* stimulation of CD8 T cells isolated from ECs with HIV-specific peptides (gag) upregulates CXCR5 (80).

The indirect cytotoxic role of these cells had been further described in autoimmune diseases. For instance, in an autoimmune hemolytic anemia murine model, Valentine et al. demonstrated a significant increase of CXCR5^+^ PD1^+^ CD8 and CD4 T cells in secondary lymphoid tissue early during pathogenesis (75). The two subpopulations upregulated ICOS, IL-21 and Bcl-6. However, treating the mice with CD8 and CD4 depleting antibodies resulted in increased survival, improved anemia, reduced B cell survival and decreased anti-erythrocyte IgG autoantibodies, suggesting the pathogenic role of these cells. Thus far, these data support the potential protective indirect cytotoxic function of T cells in the context of tumor development and viral control, while pathogenic in case of autoimmune diseases. Hence, it is important whether to harness or inhibit these indirect cytotoxic functions in the context of cancer or autoimmunity, respectively.

In line with previous studies highlighting the capability of the transcription factor Stat5 in negatively controlling CD4 Tfh cells and maintaining B cell tolerance (81, 82), Chen et al. demonstrated that the deficiency of Stat5 in CD8 T cells led to an increased autoantibody production in Ig ^HEL^ sHEL transgenic mice. This deficiency resulted in an increase in germinal center B cells and expansion of CXCR5^+^ PD-1^+^ CD8 T cell population after an acute viral infection. These data suggest that Stat5 negatively control CXCR5 PD1 CD8 T cell population as well (72). In conclusion, CD8 T cells can provide help to B cell resulting in enhancement of antibody production.

The specific cell surface molecules and cytokines expressed by CD8 T cells involved in B cell support and antibody production had been explored by Shen et al. (74). The authors demonstrated that CD8 T cells that localize to B cell follicles in tonsils and LNs express CXCR5 (74). Further, CXCR5^+^ CD8 T cells upregulate CD40L and ICOS, while polyclonal stimulation of these cells resulted in increased expression of IFNγ, IL-4 and IL-21 (74). Additionally, co-culturing TCR stimulated CXCR5^+^ CD8 T cells with autologous B cells resulted in increased production of antibodies, where this phenomenon was completely abolished by blocking either CD40L or IL-21. Finally, Loyal et al. defined a CD40L^+^ helper CD8 memory subpopulation that expresses IL-6 receptor and lacks the cytotoxicity surface marker SLAMF7 (83). Ironically, this indirect cytotoxic mechanism seems to be a double-edged sword in a way where antibodies can protect against pathogens, or kill tumor cells but also they can precipitate autoimmunity and induce a self-damage.

### 2.3 Direct non-cytotoxicity: The other face of the serial killer

Besides their known direct and indirect cytotoxic roles in host protection against wide-spectrum of pathogens and tumors, CTLs surprisingly can perform an entire array of non-cytotoxic functions using their effector molecules to protect the host. We classified these novel functions as direct non-cytotoxic since CTLs can still use their effector molecules but to protect the host against viral infections in a non-cytolytic fashion. In this section, we will discuss the studies that address these functions in the context of anti-viral and alloimmune responses.

#### 2.3.1 Anti-viral responses

##### 2.3.1.1 Human immunodeficiency virus (HIV)

**Figure 3:**
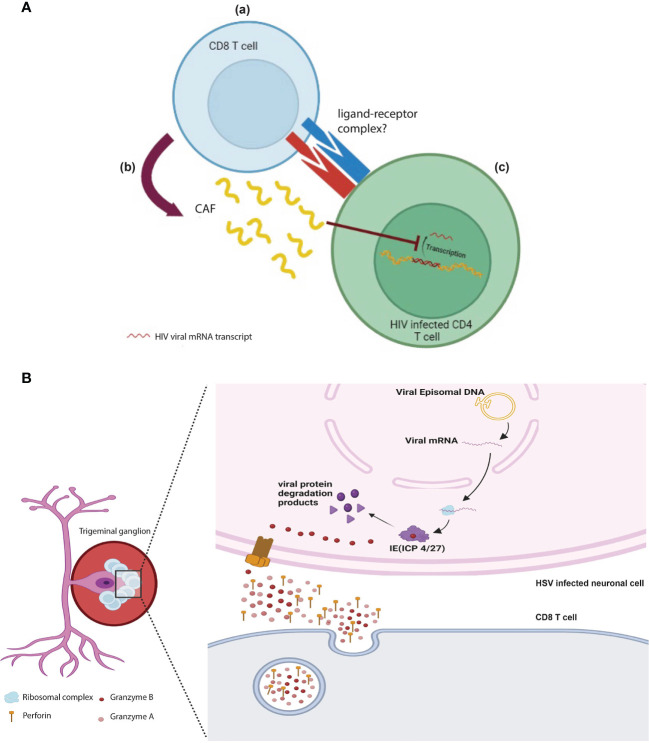
Direct non-cytotoxic effects of CD8 T cells: **(A)** HIV specific CD8 T cells (a) interact and recognize HIV infected CD4 cells *via* unknown receptor-ligand and (b) release CAF which (c) prevents the transcription and translation of HIV viral proteins and therefore prevents the propagation of viral progenies. **(B)** GzmB+ CD8 T cells cluster around the bodies of HSV-1 infected ganglionic neurons keeping the virus in latent phase by degrading viral proteins (ICP 4/27), which is essential for the viral shift to active phase. This role is mediated by perforin and granzyme B released by CD8 T cells.

The earliest report of CD8 T cell non-cytotoxicity in anti-viral immune response was first described by Waler and colleagues in 1986. In this study, the authors showed that depletion of CD8 T cells from PBMCs *in vitro* culture resulted in an increased production of HIV viral particles (84). Interestingly, this early study showed that CD8 T cells are exerting their anti-viral effect on infected cells in a non-cytolytic manner, independent of cell death, where HIV infection was kept in a dormant phase. Further work revealed that CD8 T cells’ non-cytotoxicity is mediated mostly by a secreted factor that is a protein in nature referred to as the CD8 T cell anti-viral factor (CAF) **(** **Figure 3A** **)**. The isolation of such protein is technically challenging due to its low expression profile (85).

Later on, the non-cytotoxic role of CD8 T cells has been demonstrated in non-human primates. Castro et al. reported that *in vivo* antibody depletion of CD8 T cells in AIDS associated retrovirus 2-infected chimpanzee, whose viral load is undetectable for 8 years post inoculation, leads to HIV-viremia. Nonetheless, when the animals recovered from the antibody depletion, the viral load decreased again to its initial undetectable levels (86). This study, along with other seminal early studies, demonstrated that the infected CD4 T cells were not cleared by specific CD8 T cells but rather the pro-viral DNA residing in the infected CD4 T cells was kept stable and non-transcribed (86). For more details about non-cytotoxic functions of CD8 T cells during AIDS, please refer to this excellent review (87). In conclusion, these studies along with others provide strong evidence of a non-cytotoxic anti-viral role against HIV (86, 88, 89).

##### 2.3.1.2 Hepatitis B and C viruses (HBV and HCV)

Hepatitis B-virus specific CD8 T cells play an indispensable role in controlling and resolving hepatitis B infection (90). For instance, treating a hepatitis B infected chimpanzee with CD8 depleting monoclonal antibodies at week 6 post-infection resulted in dramatic increase in the viral DNA (90), suggesting the essential anti-viral role of CD8 T cells. Although CD8 T cell cytotoxicity contributes to the viral control, this mechanism appears to come into play later in the course of the disease since viral DNA suppression preceded the peak of hepatic pathologic damage (90).

In 1994, Guidotti et al. demonstrated that CD8 T cells contribute to HBV control in a non-cytopathic-manner through inhibition of viral gene expression in transgenic mouse models (91). Since mice are inherently immune to hepatitis B infection, two elegant transgenic models were employed where they constitutively express HBV surface proteins either under the control of HBV regulatory element or under the control of murine albumin promoter (91). In this model, the administration of HBsAg-specific CD8 T cells into the transgenic mice resulted in significant reduction in hepatic viral mRNA content without induction of any hepatic damage (91). Additionally, both liver IFNγ and TNFα mRNA were elevated coinciding with hepatic CTL infiltration. To draw a cause-and-effect relationship, the authors either used IFNγ and TNFα knockout HBV transgenic mice or treated the mice with blocking monoclonal antibodies 24 hours prior to CTL injection (91, 92). In this approach, they observed failure to reduce viral mRNA. Additionally, the transfer of HBsAg-specific CTLs derived from perforin or FasL deficient mice into HBV transgenic mice clears the HBV DNA replicative forms along with the hepatocellular cytoplasmic HBV core antigen (HBcAg). These data highlight the pivotal role of IFNγ and TNFα in controlling HBV infection independent of perforin and FasL pathway (62), which raises the question: what are the molecular mechanisms underlying these functions?

It has been shown that both IFNγ and TNFα inhibit viral replication through three different molecular mechanisms: (1) upregulation of the nuclear deaminases APOBEC(A3)A and (A3)B, which resort to the hepatitis B virus core protein to get access to the covalently closed circular DNA(cccDNA), essential for viral persistence (92–94). Consequently, the deaminated cccDNA can be degraded by nucleases (92–94), (2) IFNγ can prevent the assembly of the viral RNA containing capsid in the hepatocellular cytoplasm in a proteosome and Kinase dependent-manner (95, 96), and (3) IFNγ induced proteases cleave SSB/La, an RNA binding protein that protects and stabilizes HBV mRNA, rendering the viral mRNA susceptible to endoribonucleolytic degradation (97, 98).

CD8 T cells can also play an essential role in the clearance of HCV and enhancement of protective immunity during acute infection. However, this cytotoxicity stunts with chronic infection where CD8 T cells frequently develop reduced cytotoxicity. To efficiently inhibit HCV progression, CD8 T cells develop a protective mechanism involving the TCR axis against the nonstructural protein 5 (NS5A). The NS5A TCR-specific CD8 T cells, only represent a small proportion of anti-viral CD8 T cells with a relatively low affinity requiring a higher ligand burden to initiate cytotoxicity and production of effector molecules. Nevertheless, these CD8 T cells can effectively inhibit the replication of HCV in hepatocytes keeping the HCV mRNA intact inside the infected cells. This process is rather non-cytotoxic as it does not induce a change in the level of hepatocellular enzymes such as AST (99).

The direct suppression of viral replication in HCV-infected hepatocytes can be mediated by IFNγ and TNFα, independent of cell-to-cell contact with virus-specific CD8 T cells (100). IFNγ upregulates various enzymes with robust antiviral effect such as protein Kinase R, ADAR adenosine deaminases, guanylate binding protein. These enzymes phosphorylate the eukaryotic initiation factor 2 (EIF-2), which in turn inhibits viral protein synthesis and generates truncated nonfunctional viral proteins that hinder viral replication (101). Thus far, these molecular mechanisms highlight the pivotal non-classical role of CD8 T cells in anti-HBV and HCV response. Instead of killing the virally infected cells, CD8 T cells can, *via* its effector medicators such as IFNγ and TNFα, inhibit viral replication and viral protein synthesis, limiting viral spread.

##### 2.3.1.3 Herpes simplex virus (HSV-1)

The non-cytotoxic function of CD8 T cells in anti-viral responses has been extended to involve their role in maintaining alpha herpes family infections at latency. It is widely accepted that apoptosis of virally infected or tumor cells is largely mediated by the effector molecule GzmB. However, a novel non-cytotoxic function of GzmB has been discovered by two groups in controlling the pathogenesis of human alpha herpes viruses (102, 103). In these studies, the authors showed that GzmB^+^ CD8 T cells cluster around HSV-1 latently infected trigeminal ganglia (TG) where GzmB surprisingly degrades one of the important proteins in the lytic cycle of the virus (ICP4) without induction of apoptosis **(** **Figure 3B** **).** Another study further reported additional GzmB targets expressed by HSV-1 (ICP27) and the closely related virus Varicella Zoster Virus (VZV ORF4 and ORF62). These studies highlight a novel mechanism in which CTLs prevent viral reactivation in a non-cytotoxic manner using the effector molecule GzmB (102, 103). However, it is not completely clear why GzmB^+^ CTLs did not induce apoptosis in this context. One can speculate that the viral peptides outnumbered the concentration of caspases in the cell. Hence, the peptides act as a GzmB “sponge” switching its effect away from initiation of apoptosis.

In general, the common theme in the anti-viral studies discussed in this section is the preference towards a non-cytotoxic mechanism rather than cytotoxic, which begets the question: what are the signals that drive the immune system to decide between cytotoxic vs non-cytotoxic mechanisms? The answer to this question encompasses several factors including but not limited to the type of infected tissue, degree of infection, and type of virus. For instance, the immune system might decide not to restore its cytopathic mechanisms if large number of cells are infected in the tissue specially for vital organs such as the liver or the brain. On the contrary, CTLs can eradicate virally-infected cells if they are few in number (104, 105).

#### 2.3.2 Alloimmune responses

Alloreactive memory CD8 T cells are considered as the main drivers of allograft rejection through their cytotoxic machinery (106, 107). However, Krausnick et al. demonstrated a non-cytotoxic role of CD8 T cells in regulating the alloimmune response during lung transplantation (108). In this study, the authors showed that B6 CD8 depleted mice or B6 CD8^-/-^ mice acutely reject their pulmonary allografts from Balb/c mice with a significant inflammatory infiltration in the grafted lungs. Further, the adoptive transfer of wild-type B6 CD8 T cells into immunosuppressed B6 CD8^-/-^ recipients restored tolerance to BALB/c lung allografts. The authors took their analyses one step further and performed a mixed lymphocyte reaction (MLR) to further understand the role of CD8 T cells in this model. In these analyses, they observed that CD8 T lymphocytes isolated from tolerated BALB/c→B6 lung allografts, but not spleens could inhibit proliferation of B6 congenic CD4^+^ and CD8^+^ T lymphocytes (responders) in the presence of BALB/c splenocytes (stimulators). These findings suggest that CD8 T cells with regulatory capacity accumulate in lung allografts enhancing graft tolerance, where a large proportion of CD8 T cells infiltrating tolerated lung grafts acquire an IFNγ^+^ central memory phenotype.

To further understand this phenomenon, the authors pretreated recipient mice with IFNγ–neutralizing antibody or *Ifnγ^–/–^* animals were used as hosts. Surprisingly, they observed a break in tolerance and graft rejection. Injection of *Ifnγ^–/–^* CD8 T cells into CD8^–/–^ mice failed to rescue BALB/c lung allografts from rejection, despite costimulatory blockade (108). Additionally, the authors observed that trafficking of these central memory CD8 T cells was chemokine dependent. Indeed, injection of Pertusis toxin treated CD8 central memory cells (which irreversibly inactivate Gαi-coupled chemokine receptors) into immunosuppressed B6 recipient of Balb/c lung impaired migration of central memory cells to the lung. To this end, the obvious question is: How does IFNγ act to prevent rejection? Is it a signal related to the lung microenvironment or intrinsic to the T cells? The authors took their study one step further and showed that IFNγ exerted its regulatory effect *via* a Nitric oxide (NO)-pathway. In fact, inhibition of iNOS abrogated the suppressive capacity of T cells. Hence, they showed that NO was essential in allowing graft acceptance and maintaining tolerance locally. Taken together, these data demonstrated the non-cytotoxic role of host CD8 T cells in lung allograft tolerance in an IFNγ dependent-manner. This work provided a deep insight into the role of CD8 T cells in regulating the alloimmune response in a non-cytotoxic manner, albeit such role seems to be milieu dependent as similar functions were not shown in other transplanted tissues.

### 2.4 Indirect non-cytotoxicity: The many faces of a protector

Far from cytotoxicity, CD8 T cells were shown to exhibit tissue protective functions and play an important role in healthy re-modeling in the face of inflammation (109–111). Indirectly, without resorting to their cytotoxic machinery, CD8 T cells can exert reparative functions by cross-talking with immune and non-immune cells to recruit different types of immune cells.

#### 2.4.1 Tissue repair

Early after an acute myocardial ischemic attack, lymphocytes and macrophages migrate to the necrotic myocardial area (111). Infiltrating immune cells phagocytose the necrotic debris and initiate the scar tissue formation (111). Along the same lines, Curato et al. demonstrated that a subset of CTLs play a key role in this process (110). In this study, the authors showed that CTLs expressing the Angiotensin II receptor (AT2R) are protective against myocardial ischemia through upregulation of the immunomodulator cytokine IL-10 and downregulation of the proinflammatory cytokines such as IFNγ. Further, co-culturing post-ischemic AT2R^+^ CD8 T cells with adult cardiac myocytes resulted in significantly lower apoptotic rate when compared to AT2R^-^ CD8 T cells. Adding neutralizing IL-10 antibodies to the co-culture led to an increase in the cardiac myocyte apoptotic rate in both AT2R^+^ and AT2R^-^ CD8 T cells, suggesting that IL-10 is critical for the non-cytotoxic cardioprotective effect of AT2R^+^ CD8 T cells. These findings highlighted the role of AT2R^+^ CD8 T cells in locally regulating cytokine expression, skewing it towards a reparative profile. Furthermore, the protective effect of this population was emphasized by the adoptive transfer of AT2R^+^ CD8 T cells in cardiac tissue, which reduced myocardial ischemia. Thus far, this process reduces the bystander inflammatory injury to the healthy myocardial tissue, maintains cardiac myocyte viability, and prevents one of the most drastic post infarctions sequalae, which is autoimmunity against cardiac proteins and possibly Dressler syndrome. Although the authors demonstrated the pivotal non-cytotoxic role CD8 T cells in this disease model, the means by which they are recruited to the necrotic area is still to be determined. A possible mechanism could be *via* the release of damage associated molecular patterns (DAMPs) by necrotic myocytes that might activate infiltrating macrophages, which in turn create a chemokine rich niche that helps recruitment of CD8 T cells to the site of injury.

**Figure 4:**
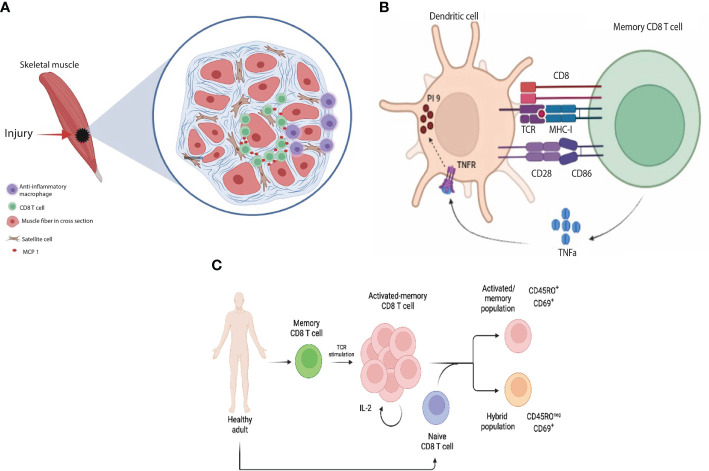
Indirect non-cytotoxic functions of CD8 T cells: **(A)** In response to skeletal muscle injury, CD8 T cells infiltrate the necrotic area and release MCP-1 which in turn helps recruit reparative macrophages to the site of injury **(B)** Upon recognition of antigen presented by DCs, memory CD8 cells release TNFα, which in turn upregulates protease inhibitor 9 (PI-9, endogenous anti-granzyme B) in DCs protecting them from CTL killing **(C)** Upon TCR stimulation, memory T cells undergo a rapid transition from a quiescent to a highly activated and proliferating state which is mediated by IL-2 cytokine downstream TCR stimulation. The cross-talk between naïve and activated memory CD8 T cells results in acquisition of two main states by naïve CD8 T cells: (1) activated/memory (CD45RO^+^ CD69^+^) and (2) hybrid population between naïve and effector (CD45RO^neg^ CD69^+^).

Later on, interest has increased to further understand the involvement of CD8 T cells in various tissue repair mechanisms. Indeed CD8 T cells have been shown to play an important role in post-traumatic skeletal muscle regeneration. Although the muscle repair process depends namely on progenitor satellite cells and anti-inflammatory macrophages, the recruitment of CD8 T cells to the inflammatory microenvironment suggests a crucial role for these cells in the regenerative process (112). Using cardiotoxin induced mouse skeletal muscle injury model, Zhang et al. demonstrated that depletion of CD8 T cells impaired skeletal muscle regeneration and increased scar formation by excessive matrix deposition (109). Consistently, adoptive transfer of CD8 T cells into CD8 knockout mice improved myofibroblast size and inhibited matrix deposition post cardiotoxin injury. CD8 knockout mice have limited recruitment of the Gr1^high^ anti-inflammatory macrophages, which are essential for skeletal muscle repair (113), into the inflammatory environment leading to a reduction in the number of satellite cells (109). CD8 T cells were further shown to play a key role in the recruitment of Gr1 ^high^ macrophages through the secretion of MCP-1 **(** **Figure 4A** **)**. The mechanism through which CD8 T cells are recruited to the injured skeletal muscle tissue is still to be identified to provide basis for a therapeutic regenerative model.

#### 2.4.2 Protection of dendritic cells (DCs)

CD8 T cells can interact with various cell types of their innate counterparts, orchestrating and fine tuning the immune response. For instance, one study demonstrated that blood circulating memory CD8 T cells, as opposed to the tissue effector CD8 T cells, have a reduced cytotoxic ability towards DCs. These cells were shown to characteristically express GzmB and perforin at a lower level. They were shown to confer a helper signal to DCs mediated by IFNγ, supporting the production of IL-12p70, a key cytokine for a Th1 immune response. Memory CD8 T cells help protecting antigen presenting DCs from the cytotoxic killing by effector T cells through the upregulation of the endogenous anti-granzyme protease inhibitor-9 (PI-9) in a TNFα dependent-manner (114) **(** **Figure 4B** **).** This provides a feedback mechanism that optimizes an effective antigen presentation and allows for a stronger immune response where potentiation of antigen presentation has a multitude of clinical implications in the area of anti-microbial and cancer vaccines.

#### 2.4.3 Homeostasis

Consciousness, the state of internal and external awareness of a living-being, remains a controversial topic among scientists and philosophers. Although it is not completely understood how conscious the immune system is, one way to explain it is through the cross-talk between wide-spectrum of immune and non-immune cells. For instance, soluble mediators such as chemokine gradients guide different immune cell types from one organ to another. Additionally, cells can crosstalk to each other through receptor-ligand interactions. Whether there is an additional means by the cells to remain conscious is yet to be discovered. Along the same lines, once memory T cells see a foreign antigen, they undergo a rapid transition to a highly activated proliferative state. Consequently, they become “conscious” of the chemokine gradient and hence, they migrate to the site of infection to clear the pathogen. For instance, TCR activation of naïve T cells results in down regulation of CCR7 and CXCR4 chemokine receptors and upregulation of inflammatory chemokine receptors including CCR3, CCR5, and CXCR3. Consequently, they acquired the capacity to migrate to inflamed tissues (115, 116). During the migration of activated memory T cells, a portion of them surprisingly migrate to antigen-free lymph nodes (117, 118) yet, the biological significance behind this route of trafficking is still enigmatic. Along the same lines, a recent study discussed the migration of unconventional T cell cells (UTCs) from peripheral tissues to draining LNs (119).

This scenario raises the question: “what is the function of memory T cells following pathogen clearance? Do they have any role(s) during homeostasis?”. One possibility could be a cross-talk between activated memory CD8 T cells and naïve CD8 T cells that continuously patrol between the periphery and LNs (120–122). Indeed, recently our lab showed that activated memory CD8 T cells acquire a novel non-cytotoxic function by which they can interact and influence the phenotype and transcriptome of naïve CD8 T cells. In this scenario, they acquire two states (1) an activated/memory T cell state and (2) a unique hybrid state between naïve and effector/activated memory cells (123) **(** **Figure 4C** **).** Since both cell populations were sorted from the same healthy subject, we speculate that activated memory T cells are presenting self-antigen to naïve T cells generating auto-reactive T cells. These findings may explain the non-cytotoxic functions of activated memory T cells and their contribution for the rise of autoimmunity following vaccination or transplantation.

## 3 Potential clinical implications of CD8 T cells’ non-cytotoxic functions

Multiple studies have discussed the non-cytotoxic anti-viral effect of CD8 T cells in clinically asymptomatic HIV-infected individuals (84, 124–127). CD8 T cells from these patients can suppress *in vitro* viral replication with CD8/CD4 T cell ratios as low as 0.05:1 (128, 129). In contrast, CD8/CD4 cell ratios, as high as 4:1, are needed to suppress 90% of the HIV replication in CD4 T cells from AIDS patients (128, 129). The mechanism of HIV replication inhibition is independent of CD4 killing since the number of CD4 in coculture with CD8 were the same as the control infected CD4 T cells alone (124, 127, 129). Further, CD8 T cells from AIDS patients demonstrated lower anti-viral activity when co-cultured with autologous, naturally infected CD4 cells or with acutely infected CD4 cells (128). This might be explained by the development of an exhausted phenotype by CD8 T cells because of persistent antigen stimulation secondary to a chronic infection. Thus, substantial differences in the CD8 T cell response between different types of HIV patients were observed (124). Regarding the elite controllers (HIV positive individuals whose immune system is capable to keep the HIV viral load under 50 copies/ml), this non-cytotoxic CD8 activity can remain stable for up to 20 years or more in some subjects not receiving anti-retroviral therapy (ART). Notably as well, the levels of integrated HIV-1 pro-viral DNA are lower in the PBMCs from clinically asymptomatic HIV-1-seropositive individuals than in progressors (86, 128). This integrated pro-viral HIV DNA increases when the CD8 T cells are depleted from their cultured PBMCs. Therefore, CD8 T cells can block the virus spread by suppressing the levels of viral mRNA as well as progeny virus.

Despite the overwhelming success of ART in controlling HIV infection, HIV-specific CD8 T cells were shown to be required for such control in tandem with ART. Indeed, *in vivo* depletion of CD8 T cells using monoclonal antibodies in 13 Indian origin SIV infected Rhesus Monkeys maintained on ART resulted in a significant increase in viral loads and SIV RNA in plasma, with minimal change in the SIV DNA containing CD4 T cells between pre and post depletion (130). This study further underlines the non-cytotoxic role of CD8 T cells in controlling viral replication. Hence, harnessing the non-cytotoxicity of CD8 T cells along with ART seems a plausible and potential therapeutic approach specially in resistant patients or progressors despite ART. As discussed previously, CAF could be a potential candidate to enhance non-cytotoxic functions of CD8 T cells in the context of HIV infection. Further, additional studies are needed to draw parallels and learn for other viral models such as HBV, HCV, and HSV-1.

Another implication for the non-cytotoxic functions of CD8 T cells is in the realm of anti-tumoral vaccination. The weak immunogenicity of tumoral antigens raised the need for a stronger immunogenic adjuvant that would confer help for anti-tumoral immune response. Bystander CD8 T cells have been shown by the previously mentioned work of Kalinski to enhance the anti-tumoral effect provided by dendritic cell-based vaccines (62). This work has been recently complemented by Newman et al. who showed that active influenza vaccine improved the outcome of lung cancer in both mouse models and human patients (131). The study further showed that intratumorally vaccination with heat inactivated influenza virus significantly reduced skin melanoma in mice and improved host survival. This effect was shown to be mediated by DCs *via* a Batf3^-/-^ mice and leds to increase of CD8 T cells and intra-tumoral anti-tumor CD8 T cells as well. Finally, CD8 T cells were shown to protect DCs (direct non-cytotoxicity) in a way to enhance antigen presentation and thereby augment the subsequent immune response (114).

In summary, enhancing the antigenicity of tumor vaccines by including a tumor non-specific antigen could be a potential therapy in addition to chemotherapy and immunotherapy. Despite these seminal studies showed a substantial help provided by such vaccines, whether this effect is mediated directly by anti-tumoral CD8 cells or anti-viral CD8 cells is still unclear. This begets the following question: do anti-tumor CD8 cells crosstalk and interact with non-specific CD8 cells in the tumor microenvironment in order to receive the needed help to amplify the anti-tumoral response?

The protective benefit of the non-cytotoxic effect of CD8 T cells can be extended to be medically employed for regeneration and tissue repair. As previously reported in this review, following an ischemic event, a subpopulation of CD8 T cells that expresses AT2R migrates to the injured cardiac tissue and participates in the post-ischemic reparative processes. This subpopulation produces IL-10 that enhances the reparative mechanisms and prevents the deleterious scar formation. It is crucial to understand the mechanisms involved in the recruitment of these cells into cardiac tissue post-ischemic injury. The first signal to recruit and activate these cells in order to initiate their reparative functions is still to be deciphered: whether it depends on an Angiotensin gradient post ischemia, DAMPs, or a specific chemokine. Identifying the first step in this cascade of events would allow finding a therapeutic measure to enhance post ischemic cardiac remodeling and prevent scar formation within the injured cardiac tissue.

## 4 Concluding remarks

CTLs are classically considered as the serial killers of the immune system. As such, they are equipped with a wide array of cytotoxic molecules such as Granzymes and perforin. They are the soldiers of the immune system that clear pathogens, and fight against tumoral growth. However, CD8 T cells assume other protective, reparative, and homeostatic roles beyond their cytotoxic capacity. Resorting to their cytotoxic molecular machinery, CD8 T cells seem to play a direct non-cytotoxic function. For instance, they were able to control infections beyond directly killing the infected cells mainly by suppressing viral replication to limit viral spread in case of HBV and HCV or maintaining viral latency (e.g., HSV-1). Further, CD8 T cells were shown to be implicated in regeneration and tissue repair especially in post-ischemic cardiac remodeling (indirect non-cytotoxicity). Understanding the non-cytotoxic functions of CD8 T cells is a critical step to harness CD8 T cell function in cancer immunotherapy and vaccines.

## 5 Outstanding questions

- Are CTLs heterogenous regarding their cytotoxic functions? Or are they plastic? In other words, can the same CTL perform both cytotoxic and non-cytotoxic functions depending on their environment and signal received?
- Why do CTLs that express GzmB do not kill HSV-1 latently infected neurons?
- In tumor vaccines, what are the mechanisms responsible for enhancement of tumor clearance? Is it crosstalk and interaction between non-specific and tumor-specific CD8 T cells? if so, how?
- How can we harness recruitment of AT2R^+^ CD8 T cells to the site of tissue injury during myocardial infarction to enhance repair? What signals are responsible for recruitment of these cells?
- Why do alloreactive T cells in lung transplantation play a protective role but not in other solid organ transplantation? What is so special about the lungs?
- What kind of signals can we learn from the lung microenvironment to apply to other solid organ transplants?
- Why is there differential non-cytotoxic capacity of CD8 T cells in HIV patients? Is it cell-intrinsic or microenvironment driven?

## Author contributions

MA help in writing the review and created the figures. HA conceive the idea, write and edit the review main text and figures. All authors contributed to the article and approved the submitted version.

## Acknowledgments

We would like to thank Srivatsa Bellamkonda for reading and helping in editing the manuscript. We would like also to thank Dr. Adrian Morelli for reading and fruitful discussion over the review (Starzl Transplantation Institute, University of Pittsburgh, School of Medicine).

## Conflict of interest

The authors declare that the research was conducted in the absence of any commercial or financial relationships that could be construed as a potential conflict of interest.

## Publisher’s note

All claims expressed in this article are solely those of the authors and do not necessarily represent those of their affiliated organizations, or those of the publisher, the editors and the reviewers. Any product that may be evaluated in this article, or claim that may be made by its manufacturer, is not guaranteed or endorsed by the publisher.
